# Soil and Climate Geographic Information System Data-Derived Risk Mapping for Grape Phylloxera in Washington State

**DOI:** 10.3389/fpls.2022.827393

**Published:** 2022-02-16

**Authors:** Abhilash K. Chandel, Michelle M. Moyer, Markus Keller, Lav R. Khot, Gwen-Alyn Hoheisel

**Affiliations:** ^1^Department of Biological Systems Engineering, Center for Precision and Automated Agricultural Systems, Washington State University, Prosser, WA, United States; ^2^Department of Biological Systems Engineering, Virginia Tech Tidewater AREC, Suffolk, VA, United States; ^3^Department of Horticulture, Irrigated Agriculture Research and Extension Center, Washington State University, Prosser, WA, United States; ^4^WSU Extension Ag and Natural Resources, Irrigated Agriculture Research and Extension Center, Washington State University, Prosser, WA, United States

**Keywords:** grapevine, phylloxera, risk prediction, soil sand content, soil temperature, geographic information system

## Abstract

Grape phylloxera (*Daktulosphaira vitifoliae, syn. Viteus vitifoliae*), a destructive root and foliar pest of grapevines, occurs in almost all viticulture regions worldwide. However, certain regions have remained “phylloxera free.” Until recently, this included Washington state (United States), where this insect is regulated as a quarantine pest by Washington State Department of Agriculture. In 2019, established phylloxera populations were discovered in Washington. Phylloxera is typically managed by using resistant or tolerant rootstocks. In Washington, most wine grapes are grown on their own roots of the susceptible species *Vitis vinifera* instead of grafted rootstock, and thus, are at high risk of vine death should they become infested with phylloxera. This article reports development of a phylloxera risk map for Washington state using geographical soil texture (sand content) and soil temperature data. Weighted averages of soil texture data (mapping year: 2016, depth: 0–100 cm) were obtained from United States Department of Agriculture-Natural Resource Conservation Service (USDA-NRCS) and soilgrids. Soil temperature data were obtained from over 200 weather stations of Washington State University’s AgWeatherNet network. Threshold-based classifications were performed in Quantum GIS software on the rasterized soil sand content and temperature independently to derive low, moderate, and high-risk areas, with risk defined as site suitability for optimal phylloxera development. The validation identified 22 out of 23 confirmed phylloxera-positive sites as “high risk,” and one site as “moderate risk” when considering soil sand content alone. Soil temperature data alone classified 10 sites as “high risk” and 13 sites as “low risk.” When soil sand content was combined with soil temperature (as a risk modifier), 10 sites were classified as “high risk,” 12 sites as “high-moderate risk” and one site as “moderate-low” risk. Ground-truth comparisons of confirmed positive sites for phylloxera agreed with past research suggesting that soil sand content is the dominant factor influencing phylloxera infestation. Pertinent risk assessment can be an important component for vineyard decision-making, including whether to use rootstocks in vineyard development or replant scenarios. It may also help to focus the initial scouting and identification efforts to sites and may be helpful when tracking and developing solutions for quarantine pests, such as phylloxera.

## Introduction

Grape phylloxera (*Daktulosphaira vitifoliae syn. Viteus vitifoliae*) is a destructive pest to grapevines (*Vitis* sp.) and is native to eastern and central North America. Phylloxera can cause damage on both leaves and roots, but it is particularly devastating as a root pest to European wine grapes (*Vitis vinifera*), as no natural resistance has evolved in that grape species (Granett et al., 2001; Powell et al., 2013; Powell and Clarke, 2018). Globally, this pest has been successfully managed through the adoption of resistant or tolerant rootstocks derived from various American *Vitis* sp. that have coevolved with the insect. However, there are a few viticulture regions that have been able to grow *V. vinifera* cultivars on their own roots due to the lack of natural incursion or human introduction of phylloxera to these regions, or because of the very high sand content of their soils (Riaz et al., 2019; Tello et al., 2019). Washington state, in the United States, was regarded as one such region and has specifically identified phylloxera as a quarantine pest in the state. The lack of native grape species in the area, and general isolation (Rocky Mountain Range), likely limited the natural distribution of the pest to this area (Callen et al., 2016). With only limited identification of the pest in the state over the last 120 years, the industry there is built on the use of own-rooted *V. vinifera* due to the relative ease that option provides when retraining vines killed after winter cold temperature events (Wolfe, 2001). However, with the identification of additional phylloxera outbreaks in 2019 (Prengaman, 2019), and subsequent confirmation of the scale of these outbreaks in 2020 and 2021, there are practical production concerns related to limiting the spread of this pest in the short-term, and whether the universal adoption of rootstocks in the state will be required for sustainable management in the long-term.

The phylloxera occurrence in a region is not always indicative of subsequent population growth and associated vine collapse. Observations have reported soil texture, specifically sand content, as the dominant influencing factor for phylloxera establishment, development, and spread. A study in South African vineyards revealed that phylloxera was predominant in soils with less than 65% sand content (De Klerk, 1972). Similar observations were reported in Canadian and Californian vineyards, where phylloxera was very common in clay, loam, and sandy-loam soils, but not in sandy soils (Nougaret and Lapham, 1928; Stevenson, 1964; Chitkowski and Fisher, 2005; Botha et al., 2007).

Soil temperature, and water content as well as the atmospheric humidity are reported to be secondary influencers of phylloxera development (Kühnelt, 1963; Helm et al., 1991; Gerson, 1996). Soil temperatures in the range of 18°C–27°C are thought to be optimal for phylloxera survival and reproduction (King and Buchanan, 1986; Turley et al., 1996; Fisher and Albrecht, 2003). These studies report that low (less than 6°C) or high (greater than 27°C) temperatures can increase phylloxera mortality and reduce reproduction rate. Temperatures in the range of 6°C–18°C seem to be more conducive for phylloxera development or spread of infestations.

When managing quarantined, or non-endemic pests, the extent of outbreaks can only be quantified through direct physical observations. Given phylloxera is a root-borne pest and symptoms of infestation may take years to manifest, these scouting efforts are laborious and regionally limiting. Management is limited to reducing additional spread of the insect through various cultural approaches, and then waiting until the vineyard has declined sufficiently to warrant replanting using resistant rootstocks (Granett et al., 2001).

However, the discovery of phylloxera within a particular region is not necessarily indicative of whether or not all vineyards in that region are at immediate risk for phylloxera infestation or damage. Thus, regional risk maps that focus on environmental favorability for development and spread, if the insect were introduced, could serve as an efficient tool to direct scouting and management decision-making efforts. These maps may also be useful for growers to assess whether or not the use of resistant rootstocks is advisable in future replants. This is the case in Washington state, where growers are trying to understand the potential future risk the current infestation may have in the region. In addition, the universal adoption of rootstocks in future vineyard plantings is not always desired, as the use of own-rooted *V. vinifera* grapevines has several economic benefits (e.g., cheaper per-plant costs, easier retraining after winter cold injury). Thus, the development of a risk map for phylloxera could be a helpful decision support tool for those concerned about this pest. Specifically, this study aimed to develop a phylloxera risk map for Washington state based on soil sand content and soil temperature data processed in geographic information system (GIS), and then validate that map by comparing the predicted risk to actual site confirmations of phylloxera infestations.

## Materials and Methods

### Soil Sand Data

**Figure 1:**
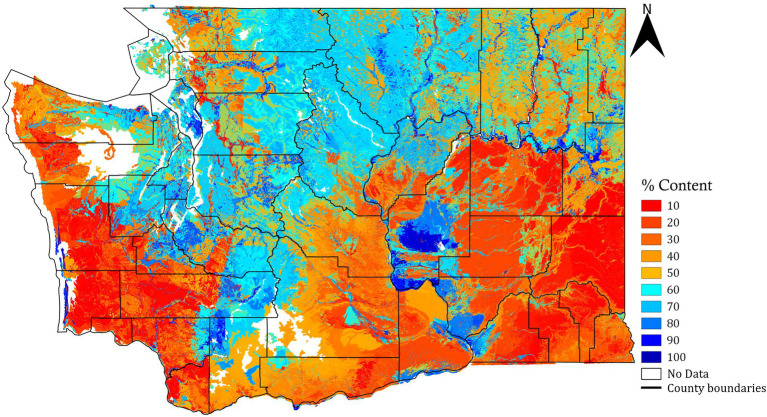
Soil sand content raster map for the state of Washington (depth: 0–100 cm, spatial resolution: 14.5 m/pixel).

Raster of soil sand content (%, weight: g of sand/100 g of soil) was obtained from the Web Soil Survey of the United States Department of Agriculture-Natural Resource Conservation Service (USDA-NRCS, year: 2016) for the state of Washington. The raster had a spatial resolution of 14.5 m/pixel and was for a depth of 0–100 cm. This raster was derived as the weighted average of sand rasters for different depths (0–5, 5–20, 20–50, and 50–100 cm). These calculations consider pixels at the soil depths where the data was available (or the soil was present) and assigns no-data values to the pixels at depth where the data was unavailable or if there was a bedrock. For the regions where the data was not available in the weighted soil sand content raster derived from NRCS, it was filled in using weighted soil sand content raster (source: https://soilgrids.org) that was also resampled for the spatial resolution of 14.5 m/pixel. The resultant soil sand raster is shown in Figure 1. There exist some areas in the resultant layer with no sand rating, possibly because of a soil restriction layer, or a soil survey was not completed in those areas.

### Soil Temperature Data

**Figure 2:**
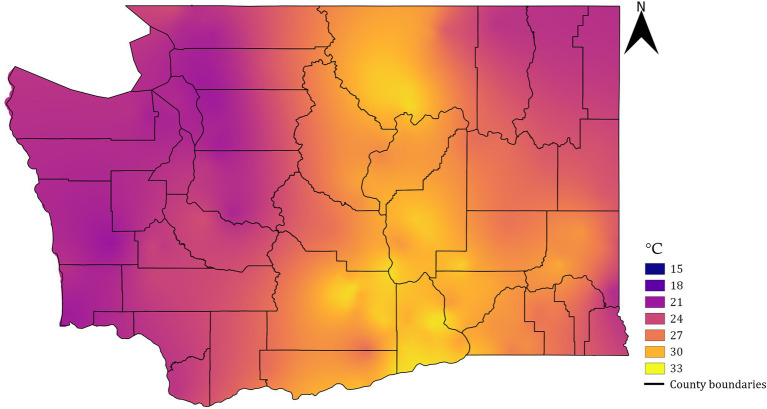
Raster map of mean daily maximum soil temperature data for Washington state within the June 1 to August 31 period averaged for 11 years (2010–2021).

Soil temperature at 20.32 cm depth, logged at 15-min interval, was acquired from all Washington State University AgWeatherNet stations (weather.wsu.edu). All stations are installed in primarily agricultural regions across the state in open field conditions following the guidelines established by the World Meteorological Organization (Gommes et al., 2010). These stations record the above-surface weather using an array of meteorological sensors and the soil temperature using a probe installed down to a depth of 20.32 cm. The application programming interface was accessed to parse the data from 200 stations spread across the state using a Python script (version 3.6.5). A date window from 1 June to 31 August from 2010 to 2021 was used to filter the data, and the mean daily maximum temperature during that 92-day period was calculated for each station. This period covers the time when temperature would likely have the biggest impact on the development rate of phylloxera (Forneck and Huber, 2009; Skinkis et al., 2009). The daily maximum temperatures were considered because the standard weather stations are often placed over the irrigated surfaces that may intermittently experience lower soil temperatures relative to actual vineyard sites. Such lowering of temperatures may lead to underprediction of the risks when considering the daily mean soil temperatures. The filtered temperature values were extracted into a “csv” file and converted to a raster layer using an ordinary kriging interpolation method in the System for Automated Geoscientific Analysis (SAGA) tool (Conrad et al., 2015) for a spatial resolution of 14.5 m/pixel. The resultant raster was clipped for the shapefile boundaries of Washington (Figure 2) using the “raster clipper tool” in Quantum GIS software (version 2.18.16, Open source project).

### Risk Prediction and Validation

**Figure 3:**
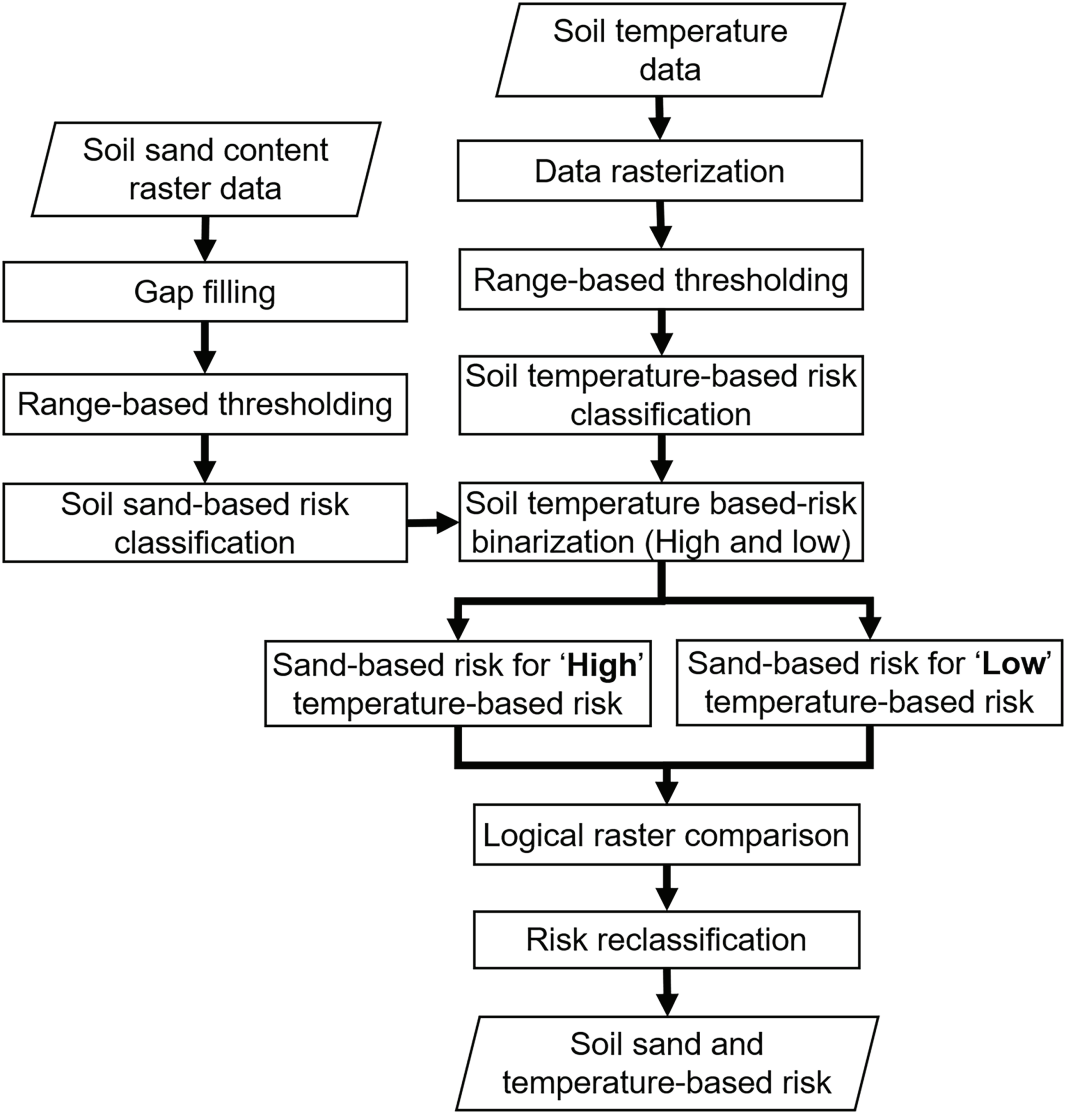
Process flowchart for mapping soil sand content and soil temperature data for assessing phylloxera infestation risk.

The process flowchart used for developing the risk maps is shown in Figure 3. All the mapping processes were conducted in QGIS (version 2.18.16, Open Source Project) and R (version 3.6.1, R Core Team, Vienna, Austria, and RStudio, Inc., Boston, MA, United States). The phylloxera infestation risk was classified based on: (1) soil sand content, (2) soil temperature, and (3) the combination of sand content and soil temperature. Sand content and temperatures were classified into risk categories by range-based thresholds (Table 1) determined from the literature based on experiments and observations (Nougaret and Lapham, 1928; Stevenson, 1964; De Klerk, 1972; King and Buchanan, 1986; Turley et al., 1996; Fisher and Albrecht, 2003; Powell et al., 2003; Chitkowski and Fisher, 2005; Botha et al., 2007). A combined risk map was developed, using both soil sand content and temperature (Table 2). For this, the sand-based risk was masked independently for temperature-based (1) low and (2) high risk masks. The resultant raster was then compared logically to modify the sand-based risk raster into five classes, as presented in Table 2. Such reclassification was based on literature findings that the soil sand content predominantly governs phylloxera development (e.g., Nougaret and Lapham, 1928). The phylloxera development risks predicted from all map inputs were validated against 23 sites within Washington state with confirmed phylloxera outbreaks that resulted in identifiable vine decline such as stunted shoot growth, and lack of fruit formation. Sites of phylloxera infestations were either visually confirmed in-person by the authors, or visually confirmed through site-collected root images with phylloxera colonies submitted by individuals associated with those vineyards. Sites are presented anonymously given the quarantine status of this pest within the state.

**Table 1:** Phylloxera risk classes based on soil sand and temperature thresholds.

Sand content (%)	Soil temperature (°C)	Risk
<65	18–27	High
65–80	–	Moderate
>80	<18, >27	Low

**Table 2:** Phylloxera risk considering combined effects of soil sand content and soil temperature, and the explanation of risk assessment for the event of phylloxera introduction to a site.

Risk			Explanation
Sand-based	Temperature-based	Overall	
Low	Low	Low	Sand content and temperature are not conducive for phylloxera development.
Low	High	Low	While sand content is not conducive, temperature is. The temperature will have an effect only if the sand content is reduced (e.g., by addition of organic matter).
Moderate	Low	Moderate-low	Sand content is moderately conducive for phylloxera development, but the temperature is not. This combination will reduce the overall risk of rapid phylloxera development.
Moderate	High	Moderate	Sand content is moderately conducive for phylloxera development, and temperatures are optimal. Phylloxera could survive and could potentially thrive if soil sand content is reduced further.
High	Low	High-moderate	Sand content is ideal for rapid phylloxera development, but soil temperature is not. Phylloxera development is possible, albeit at a slightly lower rate.
High	High	High	Soil sand content and temperatures are ideal for the rapid development of phylloxera.

## Results and Discussion

**Figure 4:**
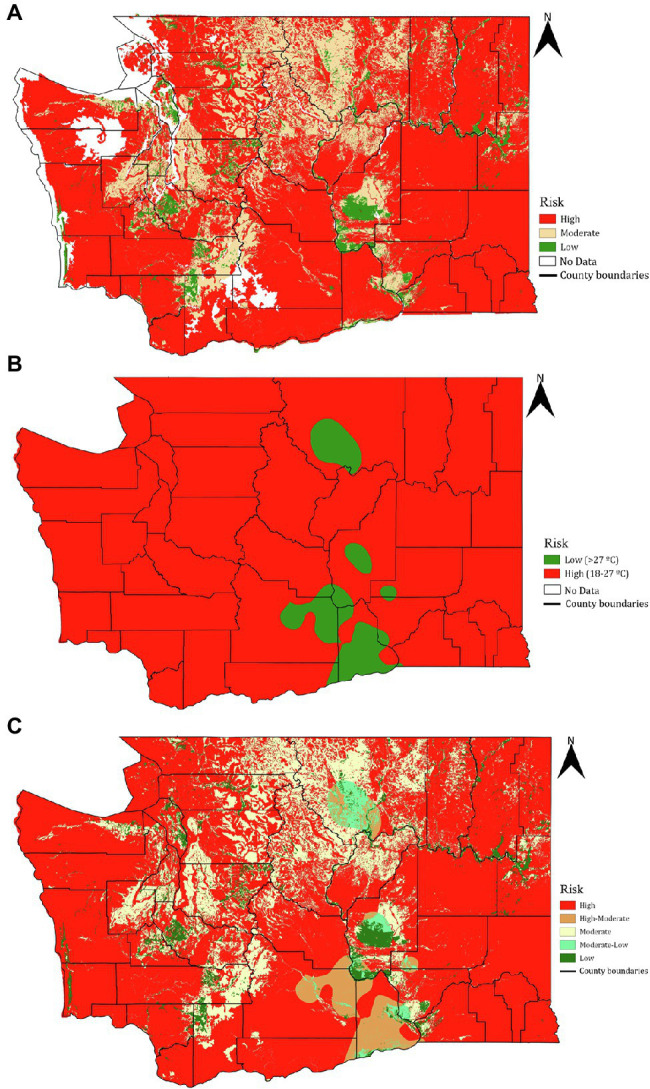
Phylloxera risk maps derived from **(A)** soil sand content, **(B)** soil temperature, and **(C)** combined sand content and soil temperature data. Larger versions of these maps are available on https://wine.wsu.edu/extension/grapes-vineyards/grape-pests/phylloxera.

**Figure 5:**
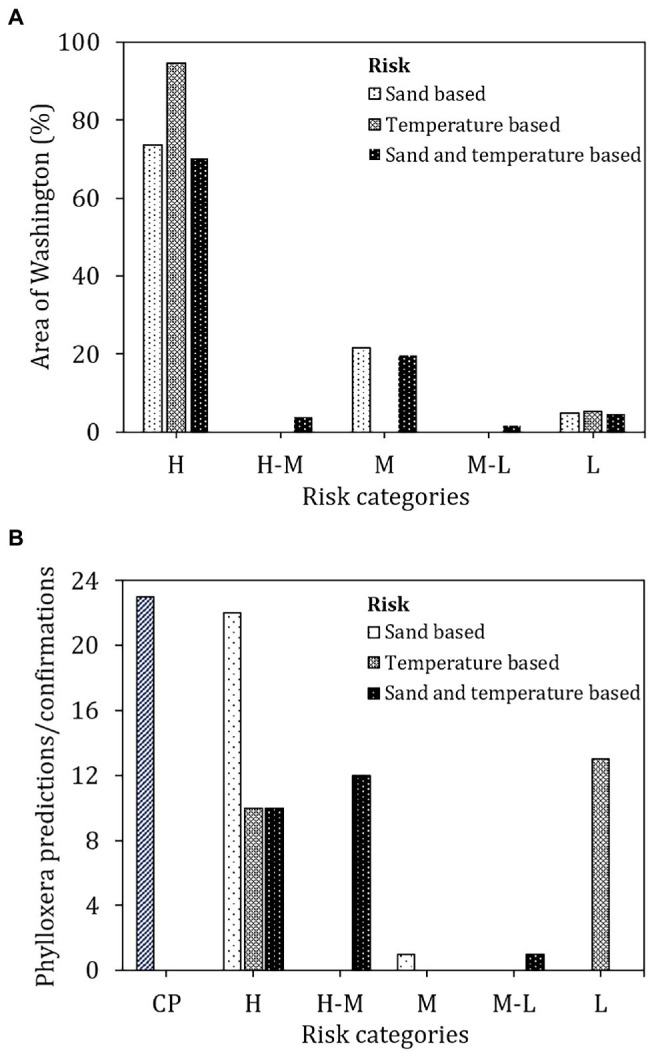
**(A)** Area under predicted risk classes and **(B)** validation with confirmed positive sites in Washington. CP, confirmed positives; H, high; H-M, high-moderate; M, moderate; M-L, moderate-low; and L, low.

The soil sand content-based risk classification (Figure 4A) identified 73.7% of the surveyed area in Washington state to be conducive for phylloxera development should it be introduced to those locations (Figure 5A). Only 4.8% of the state’s total land area is at low risk whereas 21.6% of the area was rated as moderate risk for phylloxera development. Among the 23 confirmations of phylloxera presence in the state (i.e., validation sites), 22 were identified as being in the high risk category and one in the moderate risk category (Figure 5B).

The soil temperature-based risk classification (Figure 4B) identified 94.7% of the total land area in Washington as being at high risk for phylloxera development, with only 5.4% identified as low risk (Figure 5A). Contrary to the sand-based risk assessment, the temperature-based risk identified 10 of the 23 confirmed validation sites as being high-risk and 13 as low risk (Figure 5B). The lower risks were due to the detrimental temperatures (>27°C) that would increase the phylloxera mortality (Granett and Timper, 1987; Fisher and Albrecht, 2003; Powell et al., 2003).

When soil sand content and soil temperature were combined (Figure 4C), 70.1% of the total land area was classified as high-risk, 3.9% as high-moderate risk, 19.6% as moderate risk, 1.7% as moderate-low risk, and 4.8% as low risk (Figure 5A). For the 23 confirmed validation sites, 10 were categorized as high risk, 12 as high-moderate risk, and one as moderate-low risk using this combined assessment approach.

These results demonstrate that temperature is clearly not the dominating factor influencing phylloxera development within Washington state. This conclusion is supported by prior studies in Armenia (Powell, 2012; FAO in Armenia, 2018), Russia (Afonin et al., 2009), and Australia (Powell and Clarke, 2018), where phylloxera was present at sites with extreme temperature ranges. The soil environment can provide a large thermal buffer to rapid and extreme temperature shifts, which may reduce temperature’s role as a limiting factor to phylloxera survival. For example, by reducing vertical heat conductivity, high soil moisture from rainfall or irrigation events may dampen the diurnal temperature range of a soil. By contrast, sand content was identified as the most prominent factor for phylloxera development. This interpretation is supported by studies in South African, Canadian, and Californian vineyards (Nougaret and Lapham, 1928; Stevenson, 1964; De Klerk, 1972). The underlying reasons for the observation that a high sand content prevents phylloxera development remain under debate. In addition to high sand content, high silicon in the soil has been reported as a further reducer of the phylloxera population (Ermolaev, 1990). It is also speculated that, contrasting with the well-structured clay and loamy soils, the lack of permanent soil structure in sandy soils impedes the mobility of phylloxera through soil. In addition, the coarse texture of sandy soil may result in a collapse between soil particle space and potentially increase phylloxera mortality (Nougaret and Lapham, 1928; Rombough, 2002).

In terms of other factors, studies have suggested that soil moisture content can also affect phylloxera populations, with low moisture increasing the infestation levels (King and Buchanan, 1986; Helm et al., 1991; Powell et al., 2003). Buchanan (1990) has also reported the effect of low atmospheric humidity on reducing phylloxera populations. Integration of these factors along with soil sand content and temperature could improve the risk predictions but will first need quantification of individual effects on phylloxera infestations. Currently, the best risk assessment approach appears to be the use of soil sand content as a primary predictor. Individual farms can thus improve their on-site assessments by conducting soil texture analysis of the soil within vineyard rows (i.e., under vines, or in areas where vine roots are most likely to be located), which may be warranted if soil amendments are a common practice at the site, and thus, would alter soil texture immediately around the vines.

Ultimately, the long-term management of phylloxera in Washington state, as elsewhere, will be predominately driven by the adoption of phylloxera-resistant rootstocks derived from American *Vitis* or *Muscadinia* species (Keller et al., 2012; East et al., 2021). By contrast, planting of own-rooted interspecific hybrid grapes is not recommended, because most of them contain varying levels of the susceptible *V. vinifera* in their pedigree. This principle was demonstrated by the failure in France, Italy, South Africa, and most recently California, of the hybrid rootstock Ganzin (syn. AXR#1), which has partial *V. vinifera* ancestry (Riaz et al., 2019). However, the availability of risk maps such as the ones presented here can provide a useful early-intervention tool for individuals planning new vineyard developments or facing replanting decisions, regardless of the known status of phylloxera at their future or current vineyard. For example, even if phylloxera is not present at their particular site, but the site falls into a moderate or high risk category, a grape grower may choose to adopt the use of rootstocks during vineyard establishment or replanting to avoid future risk of vine loss. Conversely, if a vineyard is in an isolated location and is in an area of low risk, the grower may choose to continue to use own-rooted vines, as cold damage during winter might pose the greatest risk for limiting production (Ferguson et al., 2014), for which own-rooted vines might be better suited for that risk mitigation.

Overall, regional risk maps could be used by extension specialists and growers to improve or focus scouting efforts, which is the only true way of determining whether phylloxera is present at a site. This risk map (and Table 2) also provides decision support for longer-term management decisions, such as the use of rootstocks in the case of phylloxera management. Similar GIS data inputs could also be used to develop regional risk maps for other insect/pest/disease infestation for various other horticultural crops and in other regions.

## Conclusion

The GIS inputs of soil sand content and soil temperature could estimate the risk of phylloxera development in Washington state following introduction of the insect to a vineyard site. As individual inputs, soil sand content provided the most accurate risk assessment when compared to confirmed locations (validation sites) with phylloxera, where 100% of the validation sites were classified as either high risk or moderate risk, and 96% of the validation sites were classified as high risk. Soil temperature, as an individual input, was not as predictive, classifying only 43.5% of the validation sites as high risk. When combined, 95.7% of the validation sites with known phylloxera presence were classified as high or high-moderate risk, and 4.3% of the validation sites were classified as moderate-low risk. While further investigation on weighting factors for combining different variables (soil sand, soil temperature, soil moisture, atmospheric humidity, etc.) could improve the risk predictions, using soil sand content appears to be an excellent predictor based on our results. Site-specific (vineyard) data related to soil management and modifications could further improve the risk predictions.

## Data Availability Statement

The raw data supporting the conclusions of this article will be made available by the authors, without undue reservation.

## Author Contributions

AC, G-AH, MM, MK, and LK: conceptualization, risk validation, and visualization. AC: data collection, processing, analysis, and writing—original draft preparation. G-AH, MM, MK, and LK: resources, supervision, project administration, funding acquisition, and writing—review and editing. All authors contributed to the article and approved the submitted version.

## Funding

This study was supported by the Washington State Grape and Wine Research Program and the USDA National Institute of Food and Agriculture, Hatch project 1016563.

## Conflict of Interest

The authors declare that the research was conducted in the absence of any commercial or financial relationships that could be construed as a potential conflict of interest.

## Publisher’s Note

All claims expressed in this article are solely those of the authors and do not necessarily represent those of their affiliated organizations, or those of the publisher, the editors and the reviewers. Any product that may be evaluated in this article, or claim that may be made by its manufacturer, is not guaranteed or endorsed by the publisher.
