# Supplementary material for: Soil and Climate Geographic Information System Data-Derived Risk Mapping for Grape Phylloxera in Washington State
Source: Front Plant Sci. 2022 Feb 16;13:827393. doi: 10.3389/fpls.2022.827393 (PMC8888419; doi:10.3389/fpls.2022.827393)
Supplement: Supplementary file 1 [file Data_Sheet_1.docx]

**R code for phylloxera risk classification based on sand content, soil temperature, and both**

#Created by: Abhilash Chandel

setwd("D:") # Set the working directory #

# Include the libraries to work with the raster layers #

library(raster)

library(rhdf5)

library(rgdal)

library(sf)

# Load the sand raster layer for source 1 #

Sand1 <- raster ("WA_Soil_Sand_WMean_0_100cm_s1.tif")

# Load the sand raster layer for source 2 #

Sand2 <- raster ("WA_Soil_Sand_WMean_0_100cm_s2.tif")

# Resample the source 2 sand raster to source 1 sand raster #

Sand2_r <- resample(Sand2, Sand1, method= "ngb")

# Fill the missing values in Sand1 using Sand2_r #

Sand <- cover(Sand1, Sand2_r)

# Load the soil temperature raster layer #

SoilTemp <- raster("WA_Soil_Temp.tif")

# Resample the soil temperature raster to sand based raster #

SoilTemp <- resample(SoilTemp, Sand, method= "ngb")

# Define risk threshold classess for sand content (1: High, 2: Moderate, 3: Low) and convert to matrix form #

class_sand <- c(0,65,1,

65,80,2,

80,100,3)

rcl_sand <- matrix(class_sand, ncol=3, byrow=TRUE)

# Define risk threshold classess for soil temperature (2: High, 3: Low) and convert to matrix form #

class_soil_temp <- c(18,27,2,

27,30,3)

rcl_soil_temp <- matrix(class_soil_temp, ncol=3, byrow=TRUE)

# Phylloxera risk raster based on sand content #

Risk_sand <- reclassify(Sand, rcl_sand)

# Save the risk classified raster #

writeRaster(Risk_sand, filename="WA_Phyrisk_Sand.tif",format="GTiff", options="COMPRESS=LZW", overwrite = TRUE, NAflag = -9999)

# Phylloxera risk raster based on soil temperature #

Risk_soil_temp <- reclassify(SoilTemp, rcl_soil_temp)

# Save the risk classified raster #

writeRaster(Risk_soil_temp, filename="WA_Phyrisk_SoilTemp.tif",format="GTiff", options="COMPRESS=LZW", overwrite = TRUE, NAflag = -9999)

### Combined risk classification ###

# Sand risk raster masking based on soil temperature risks (High and Low)

Rfhigh <- Risk_sand[Risk_soil_temp = 2]

Rflow <- Risk_sand[Risk_soil_temp = 3]

# Create a dummy raster with sand risk raster corresponding to high soil temperature risk #

r3 <- raster(Rfhigh)

# Fill the dummy raster based on comparison of above masked rasters (Resultant values; 0: No risk, 1: High, 2: High-Moderate, 3: Moderate, 4: Moderate-low, 5: Low) #

# Fill where no values exist in Rfhigh #

r3[Rfhigh == 0 & Rflow == 0] <- 0

r3[Rfhigh == 0 & Rflow == 1] <- 2

r3[Rfhigh == 0 & Rflow == 2] <- 4

r3[Rfhigh == 0 & Rflow == 3] <- 5

# Fill where no values exist in Rflow #

r3[Rfhigh == 1 & Rflow == 0] <- 1

r3[Rfhigh == 2 & Rflow == 0] <- 3

r3[Rfhigh == 3 & Rflow == 0] <- 5

# Save the risk classified raster #

writeRaster(r3, filename="WA_Phyrisk_Combined.tif",format="GTiff", options="COMPRESS=LZW", overwrite = TRUE, NAflag = -9999)
